# Supplementary material for: Evaluation of radiation treatment volumes for unknown primaries of the head and neck in the era of FDG PET
Source: PLoS One. 2020 Apr 10;15(4):e0231042. doi: 10.1371/journal.pone.0231042 (PMC7147765; doi:10.1371/journal.pone.0231042)
Supplement: Attachment — Submitted filename: Comment-response.docx [file pone.0231042.s001.docx]

Comment-response:

Reviewer #1: The authors presented the outcome comparison study between unilateral and bilateral irradiation of head-and-neck cancer. Two centers’ data were collected and compared. They found no significant difference between two treatment schemes, and thereafter suggested preference towards unilateral irradiation. This study did not add too much novelty in addition to published results (reference 8-13). More importantly, there are so many confounding factors in this comparison which were not well controlled or discussed. As described in the method, patients received different chemo, surgery options, target delineation guideline, radiation dose prescription, radiation treatment modality (3D vs IMRT). All these factors would contribute to the final OS comparison, which makes the conclusion that both treatment schemes resulted in similar OS less convincing. There are substantial amount of grammar errors throughout the manuscript which hinders reading and understanding. I do not recommend the manuscript to publish as it stands. More details are as follows.

The authors need to add line number in the manuscript to facilitate review process.

Thank you for this recommendation. This has been addressed.

In the abstract under methods section, “patient factors” were not clearly explained.

Thank you for this recommendation. This has been addressed (see line 34)

In the abstract under result section, check the grammar of the first sentence.

Thank you for this recommendation. This has been revised (see line 38)

The abstract did not reflect if this study has anything to do with FDG-PET, which is listed in the title.

Thank you for this recommendation. This has been addressed (see line 28)

In material and methods section third paragraph, the authors described center B target delineation as “Target volume delineation and prescription dose was at the discretion of the treating physician based on patient and tumor factors”. The target delineation and dose prescription variation adds to the uncertainty of the outcome comparison between unilateral and bilateral irradiation. Since all these clinical factors are not well controlled, the finding of outcome comparison is less convincing.

It is difficult in this retrospective study to control for many clinical factors, particularly given the rarity of this scenario and the large span of time of which these patients were collected. As such, target volume delineation would likely be influenced by the patient’s clinical presentation/history and physician preference, which is hard to standardly describe in methods.

Also under material and methods section, a key information is missing. The authors did not provide rationale of the choice between unilateral and bilateral irradiation. This decision could be largely decided by clinical evidences, which could also be a confounding factor in the outcome analysis. It is also interesting that center A has preference towards unilateral irradiation while center B showed opposite preference.

A clear, non-controversial indication for bilateral neck irradiation is disease involvement of bilateral necks. In the absence of clinical or radiographic evidence of bilateral disease, it is a matter of physician preference on whether to include the elective neck. Factors which may have influenced this decision such as nodal burden of the involved neck, where well balanced between both centers (see table 2, nodal stage). As such, the reason for the differences between the centers likely lies within treatment philosophy (conventional bilateral neck vs unilateral) and is the subject of this manuscript. While there may be confounding factors when comparing two different centers, previous attempts to address this in a prospective randomized fashion failed to accrue.

For both centers, all patients received PET imaging but how the imaging evidence was used is not described.

Thank you for this comment. Clarification has been added on line 94

In discussion, as the authors listed, the current study discovered similar finding as in reference 8-13. I wonder what the novelty or extra finding is from this study.

Thank you for this comment. The novelty and importance of conducting this study in the PET-era has been discussed and/or clarified on lines 71-74, 144-163, 185-189

In conclusion, the authors claimed that unilateral irradiation would have decreased toxicity, which is not supported by any data in this study.

Thank you for this comment. There is no data in this study to support this statement, however previous studies which are cited after this comment have shown reduced rates of mucositis/xerostomia with unilateral neck irradiation (lines 171-184)

Both figures are poorly produced. In the vertical axis caption, there is wavy line underneath.

Thank you for identifying this. We have removed the lines in each figure.

Reviewer #2: The manuscript entitled “Unilateral irradiation for unknown primaries of the head and neck in the era of FDG-PET” describes the evaluation of the effects of radiation treatment volume on recurrence and mortality among head and neck cancer patients with unknown primaries who were treated at two different cancer centers. So the study concludes unilateral radiation therapy compared with bilateral produced to have similar survival.

General Critique:
I would like to raise the following points:
There is a major problem with the manuscript which might decrease the enthusiasm of this review. From a general standpoint, the manuscript describes clinical presentation and outcome in occult primary H & N patients and recruited cases from two referred cancer hospitals of New York retrospectively. All patients had confirmed squamous cell carcinoma (SqCC), and received routine PET scan and direct examination, and which unable to detect the origin of primary. However, PET detection rates of 29-54% have been reported in the detection of occult primary of H and N SqCC in the literature. Greater than 90% of unknown primary designations (lymph nodes in patients with no detectable primary) reflect HR‐HPV–associated OPX cancer in the current literature since 2010. Moreover, a large majority of NPC are positive for EBER on ISH. So p16 immunohistochemistry and EBER are recommended for all cervical lymph nodes with carcinoma of unknown primary site and adopted by new AJCC staging system since 2016. The data of P-16 and EBER should be included in the study. In the clinical scenario, there is lack of clinical significance only based on PET and direct examination to allocate the patients as occult primary.

Thank you for your comments. As these patients were treated from 2000-2015, routine HPV/p16 testing was not performed in the majority of patients. While this is a limitation to this study, this is an unfortunate function of attempting to collect enough patients to adequately study a fairly rare clinical scenario.

Per the NCCN, while HPV positivity may guide RT to oropharyngeal only, EBV testing alone may not be sufficient to direct treatment.

Specific concerns:

1. The section of Results should be stated more clearly and detailed clinical information including radiation volume, chemotherapy regimen, and type of operation.

Thank you for this comment. We have provided an additional “table 1” outlining the different treatment volumes, modalities, and concurrent therapy by center.

2. The assessment of score of radiation toxicity and quality of life should be included.

This was not routinely collected during the time of treatment however previous studies (refs 9, 13, 15) have documented worse toxicity (mucositis/xerostomia) with bilateral neck treatment.

3. The quality of the tables, figures needs to be improved.

Thank you for identifying this. We have corrected the wavy lines in each figure.
